# Supercritical Antisolvent Processing of Nitrocellulose: Downscaling to Nanosize, Reducing Friction Sensitivity and Introducing Burning Rate Catalyst

**DOI:** 10.3390/nano9101386

**Published:** 2019-09-27

**Authors:** Oleg S. Dobrynin, Mikhail N. Zharkov, Ilya V. Kuchurov, Igor V. Fomenkov, Sergey G. Zlotin, Konstantin A. Monogarov, Dmitry B. Meerov, Alla N. Pivkina, Nikita V. Muravyev

**Affiliations:** 1 N.D. Zelinsky Institute of Organic Chemistry, Russian Academy of Sciences, Moscow119991, Russia; oleg_dobrynin@list.ru (O.S.D.); m.n.zharkov@gmail.com (M.N.Z.); kuchurov@mail.ru (I.V.K.); ootx@ineos.ac.ru (I.V.F.); zlotin@ioc.ac.ru (S.G.Z.); 2 N.N. Semenov Federal Research Center for Chemical Physics, Russian Academy of Sciences, Moscow 119991, Russia; k.monogarov@gmail.com (K.A.M.); mmeerov@mail.ru (D.B.M.); alla_pivkina@mail.ru (A.N.P.)

**Keywords:** nitrocellulose, supercritical antisolvent process, nanoparticles, combustion

## Abstract

A supercritical antisolvent process has been applied to obtain the nitrocellulose nanoparticles with an average size of 190 nm from the nitrocellulose fibers of 20 μm in diameter. Compared to the micron-sized powder, nano-nitrocellulose is characterized with a slightly lower decomposition onset, however, the friction sensitivity has been improved substantially along with the burning rate increasing from 3.8 to 4.7 mm·s^−1^ at 2 MPa. Also, the proposed approach allows the production of stable nitrocellulose composites. Thus, the addition of 1 wt.% carbon nanotubes further improves the sensitivity of the nano-nitrocellulose up to the friction-insensitive level. Moreover, the simultaneous introduction of carbon nanotubes and nanosized iron oxide catalyzes the combustion process evidenced by a high-speed filming and resulting in the 20% burning rate increasing at 12 MPa. The presented approach to the processing of energetic nanomaterials based on the supercritical fluid technology opens the way to the production of nitrocellulose-based nanopowders with improved performance.

## 1. Introduction

Nitrocellulose (NC) is widely used as a base substance for conservation, adhesives, membranes and coatings [1,2,3,4,5] with an important application for solid rocket propellants and gunpowder production [6,7,8]. Long-term experience with nitrocellulose reveals some of its limitations, including certain thermal and mechanical hazards [9], inhomogeneity problems resulting from the organic nature of the cellulose precursor [10], and poor combustion performance. These challenges can be addressed by downscaling nitrocellulose to nanosized powder that should improve the burning rate, as was reported for another energetic substance, 1,3,5-trinitro-1,3,5-triazinane (RDX), whose nanoparticles demonstrate a twofold burning rate increase [11]. Also, the available data for the nanosized energetic materials show that their mechanical sensitivity is often beneficially reduced [12,13,14]. To date, several examples of NC with nanosized morphology have been reported. Electrostatic spinning of NC solution results in the formation of fibers with diameters from 80 to 300 nm [15,16]. The samples demonstrate the slight lowering of the decomposition onset from 198 °C to 190 °C [16], while the heat of reaction was increased [15] for nanosized NC as compared to as-received material. A complementary use of the sol-gel approach and sc-CO_2_ drying allows obtaining the NC aerogel with 30–50 nm particles that also show a lower decomposition onset temperature compared to raw NC [17]. Besides, the thermolysis mechanism of this porous material is altered by its increased adsorption capacity. Zhang and Weeks explored the low temperature solvent evaporation technique to prepare nearly 500 nm-sized NC spheres (5 °C, dimethylformamide) and thin films of submicron NC (5–45 °C, MeOH or EtOH) [18]. Notably, the obtained films exhibited a more complete combustion with the rate 350% higher than that for films of ordinary nitrocellulose. From the chemical engineering prospective, there are definite complications related to the reported preparative techniques, i.e., physicochemical methods (e.g., anti-solvent [19,20], suspension dispersion [21], recrystallization [18]) are inevitably accompanied by the issues of wasted organic solvents utilization and removal of their traces trapped inside the product, whereas mechanical methods (e.g., milling [22]) are incompatible with the polymeric nature of NC. Thereby, the challenge to develop a ‘greener’ and more efficient approach for a NC morphology modification has not been addressed yet.

Tailoring the burning rate of the double-based propellants, i.e., nitrocellulose-based formulations, is usually performed by adding of 1–10 wt.% of catalysts or inhibitors [23,24]. The general conclusion drawn on the basis of the experience with catalysis of double-based propellants is that carbonaceous skeleton formed above the burning surface promotes the burning rate via the prolongated residence of catalyst particles and increase of heat flux to the condensed phase [24,25,26]. The desired carbonaceous layer can be formed either during combustion of some components, e.g., dinitrotoluene, dibutyl phthalate plasticizers, or by introduced prior to combustion additives (e.g., 0.5–3 wt.% of carbon black in [24]). Interestingly, the addition of carbon in the form of carbon nanotubes (CNTs) can increase the burning rate per se, by improving the thermal conductivity [27,28,29]. Thus, in the recent studies [30,31] the optimal level of multiwall carbon nanotubes additive was found to be about 1 wt.%, whereas at higher loadings the combustion is depressed. Nano-sized metal oxides render the catalytic action in diverse processes, including the decomposition and combustion of energetic materials [32,33,34]. Specifically, nano-Fe_2_O_3_ has been found to catalyze the decomposition of NC, with the governing effect of the contact surface between two components [35]. Therefore, improvement of the nitrocellulose combustion requires the composites designed to deliver the nano-sized additives to the reaction zone.

Recently, sub- and supercritical fluids media have proved valuable for modification and synthetic applications in chemical engineering [36,37]. Particularly, supercritical carbon dioxide (sc-CO_2_) is most attractive due to its low critical point (P_c_ = 7.38 MPa and T_c_ = 31.0 °C [38]), non-toxicity and environmental friendliness [39,40]. Supercritical Anti-Solvent (SAS) applications of CO_2_ are based on its very good miscibility with the majority of organic solvents and insolubility of the target substrate in resulting binary solvent systems (sc-CO_2_/solvent) that causes the precipitation (recrystallisation) of the final material [41,42]. The SAS technology has already been applied for micronization of nitramine explosives [43,44]. Herein, we report the first application of the supercritical antisolvent process to prepare the uniform nano-sized nitrocellulose powder. Furthermore, NC-based composites with carbon nanotubes and nano-Fe_2_O_3_ as the burning rate modifiers have been also fabricated via the SAS processing. Thermal behavior, mechanical sensitivity, and combustion performance were evaluated showing the enhanced burning rate and reduced sensitivity for prepared nano-nitrocellulose and its composites.

## 2. Materials and Methods 

### 2.1. Materials

**Figure 1:**
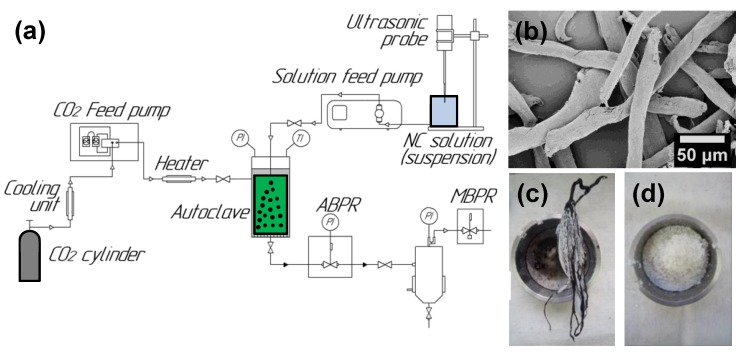
Supercritical anti-solvent (SAS) fabrication of nitrocellulose (NC)-based composites: (**a**) scheme of the experimental setup, (**b**) scanning electron microscopy (SEM) of raw NC fibers, (**c**) product obtained under non-optimized conditions, (**d**) pure NC after SAS treatment in optimized conditions.

Commercial nitrocellulose was used as a precursor for synthesis and as a reference material. The powder is constituted by fibers with a characteristic diameter of about 20 μm, as shown in Figure 1b. The elemental composition of the raw NC was found to be 26.7 ± 0.2 wt.% C, 2.9 ± 0.1% H, and 11.5 ± 0.1% N. Multi-walled carbon nanotubes (“Taunit-M” trademark by NanoTechCenter Ltd., Tambov, Russia) were used as-received. In line with the manufacturer specification [45] the inner diameter of CNTs was ca.10 nm, the outer diameter near 20 nm, and the length no less than 2 μm (Figure S1c,d, see Supporting information for details). The laboratory sample of nanosized α-iron oxide was produced by the plasma condensation technique and appeared as spherical particles 0.05–0.3 μm in diameter (Figure S1e,f, Supporting information). High purity carbon dioxide (Russian standard TU 2114-006-05015259-2014, Linde Gas Rus, Ltd., Balashikha, Russia) and acetone (Russian standard TU 2633-012-29483781-2009, Chimmed Ltd., Moscow, Russia) were used during the study.

### 2.2. Fabrication of Composites

Figure 1a illustrates the scheme of the experimental setup for the composite fabrication with the key units indicated. Cooled CO_2_ was transferred from the cylinder through the high-pressure pump into the 500 mL autoclave reactor with temperature-control. The pressure inside the autoclave was maintained with the automated back pressure regulator (ABPR). The NC acetone solution (2 wt.%) or suspension of selected fillers in this solution was fed with another pump to the filled with sc-CO_2_ autoclave through the capillary in the lid. The mutual diffusion of CO_2_ and acetone results in the precipitation of NC or its composite particles which were collected on the metallic filter at the bottom. The binary mixture CO_2_-acetone left the autoclave, went through ABPR and was divided inside the separator at the lower pressure controlled with the manual back pressure regulator (MBPR). The main units of the setup were connected with 1/8” steel pipes.

The general procedure implies filling with CO_2_ and pre-heating the system up to 40 °C. Once the desired temperature was achieved, the carbon dioxide feeding rate was set at 50 g·min^−1^. NC solution or suspension, prepared and maintained by ultrasonic vibrations, was fed at 1–2 mL·min^−1^ rate. After the solution/suspension was fully transferred to an autoclave, and CO_2_ flow pumped for an extra 30–40 min to flash off the residual acetone. Finally, the system was decompressed, and ~1 g of the target solid was taken out.

## 3. Results

### 3.1. Optimization of the Process Conditions

Preliminary runs resulted in the “herring-bone” structures instead of the desired uniform powder, as shown in Figure 1c. Several parameters of SAS process were tuned to improve the product morphology, i.e., the autoclave pressure, the ratio between the feeding rates of the solution and that of CO_2_ (f/F), NC concentration in the solution, and the additive material type (Section S3, Supporting information). Pressure variation within the 9–15 MPa range specifies the optimal value to be 10–12 MPa. At lower pressures the “herring-bone” was observed, whereas at higher pressures the uniform powder was obtained (cf. Figure 1c,d). The factor f/F controlled both the production time and CO_2_ consumption; however, its increase was shown to worsen the composite morphology. Thus, the solution flow rate appeared to be no higher than 2 mL·min^−1^ at the maximum CO_2_ feeding rate of 50 g·min^−1^ given by the experimental setup. The nitrocellulose concentration in the solution apparently determines the acetone and CO_2_ consumption and has to be maximized. On the other hand, its increase above 2 wt.% had adverse effects on the the NC dissolution time and the pump performance (due to an increase in the mixture viscosity). When the filler, i.e., and/or nano-Fe_2_O_3_, was introduced into the solution, the ultrasonic processing (30% of 70 W, 20 kHz) was applied continuously to prevent settling and to maintain the suspension shelf life.

### 3.2. Nano-Nitrocellulose and Composites 

**Figure 2:**
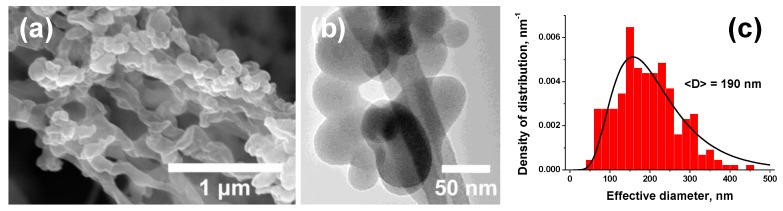
Morphology of *NC (sc)*: scanning (**a**), transmission (**b**) electron microscopy, and numerical particle size distribution (**c**). Note that image (b) shows the nanoparticles localized on transmission electron microscope (TEM) grid.

Once the SAS process parameters were optimized, four samples were obtained: *NC(sc)* pure nitrocellulose after SAS, *NC/CNT(sc)* nitrocellulose with 1 wt.% of carbon nanotubes, *NC/Fe_2_O_3_(sc)* nitrocellulose with 5 wt.% of nano-iron oxide, *NC/CNT/Fe_2_O_3_(sc)* nitrocellulose with 1 wt.% of CNTs and 5 wt.% of nano-Fe_2_O_3_. The elemental composition of *NC(sc)* was unaffected by the processing: 26.7 ± 0.1 wt.% C, 2.9 ± 0.4% H, and 11.6 ± 0.1% N. In contrast to the large fibers of initial NC (Figure 1b), the product after the SAS processing with no additives appeared as nanopowder. Electron microscopy revealed that the microstructure of the powder was formed by the nano-sized spheres of 50–450 nm in diameter that were located on thin polymeric fibers (Figure 2a,b). Numerical particle size distribution gave the average diameter of 190 nm (Figure 2c, Section S4, Supporting information). Note, that transmission electron microscope (TEM) images have captured a sole fraction that is below 100 nm. Apparently, the bigger fractions were lost during the sample preparation. 

**Figure 3:**
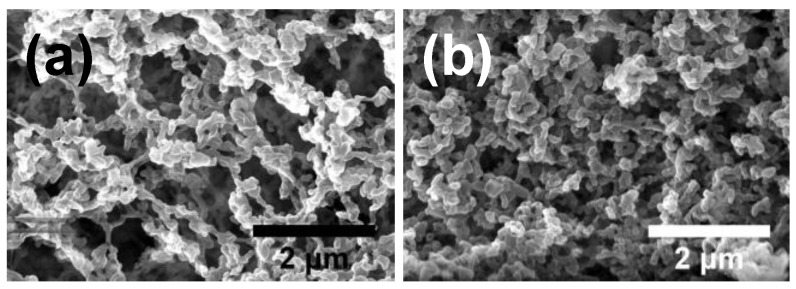
Electron microscopy of (**a**) NC/carbon nanotube (CNT)(sc) and (**b**) *NC/CNT/Fe_2_O_3_(sc)*.

With the introduction of nanotubes the skeletal structure becomes more pronounced (Figure 3a). However, the further addition of nano-Fe_2_O_3_ resulted in the material with uniformly distributed nanoparticles and with no evidence of webbing (Figure 3b). These must have been the nanoparticles of iron oxide that promote the formation of NC nanospheres instead of fibers. Notably, the catalyst content within the fabricated composites was close to the target value, i.e., no material is lost during preparation (Section S5, Supporting information).

### 3.3. Thermal Behavior

**Figure 4:**
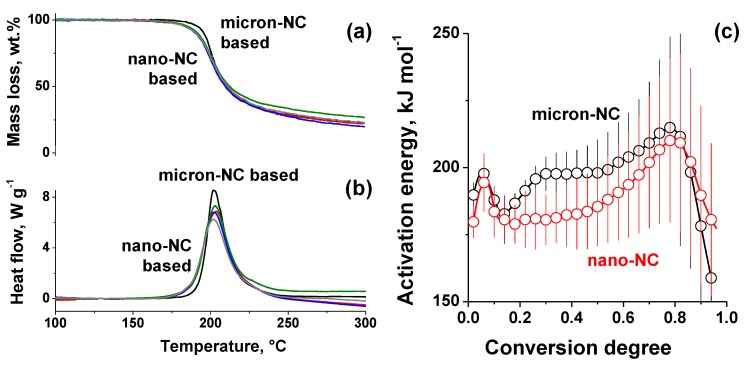
Thermal analysis under linear heating: mass loss (**a**), heat flow (**b**) data, and isoconversional kinetic analysis results (**c**).

The linear heating of all samples shows the general pattern consistent with the typical nitrocellulose behavior [46,47]: a single peak of the heat release started at about 190 °C with the corresponding mass loss of nearly 80 wt.%. The heat released during thermolysis was within the range of 1800–2000 J·g^−1^ and agreed closely for all analyzed species within the experimental error. A closer examination of the four obtained nano-NC-based samples showed a lower decomposition onset temperature (see Figure 4 and Table 1), previously observed for nitrocellulose with nano-sized features [16,17,18]. The isoconversional Friedman analysis showed that the activation energy remained the same as for micron-NC at low- and high-conversion degrees, while at the medium range the barrier was notably lower for nano-NC (Figure 4c). A similar conclusion is drawn from the formal kinetic fit with a single reaction model in flexible reduced Sestak–Berggren form [48,49]. Kinetic parameters for nano-NC, *E*_a_ = 186 ± 1 kJ·mol^−1^ and ln *A* = 43.0 ± 0.2 s^−1^ were lower than that for raw NC, *E*_a_ = 196 ± 1 kJ·mol^−1^ and ln *A* = 45.7 ± 0.3 s^−1^ (Section S6, Supporting Information). Both pairs of the kinetic parameters were consistent with the global autocatalytic reaction proposed by Brill and Gongwer [50]. The shift of the kinetic parameters for nano-NC was apparently caused by the higher surface area available for autocatalytic agents and its increased contribution to the overall decomposition process.

### 3.4. Friction Sensitivity 

The full set of experiments according to standard STANAG 4487 [51] have been performed to derive the friction force corresponding to the 50% probability of explosion. The SAS processing decreased the pure nitrocellulose *NC(sc)* sensitivity, and the required initiation stimulus accordingly rose from 234 to 342 N. The effect must have been caused by the particle size reduction since the chemical composition of the sample was unaltered as shown above. The addition of the iron oxide sensitized the *NC/Fe_2_O_3_(sc)* sample down to the level of untreated raw compound. Introduction of carbon nanotubes ranked the composite *NC/CNT(sc)* as friction insensitive (10% of explosions at highest load of 360 N) and with the addition of both carbon nanotubes and iron oxide *NC/CNT/Fe_2_O_3_(sc)* the effect was still observed (40% of explosions at 360 N).

### 3.5. Combustion Tests 

**Figure 5:**
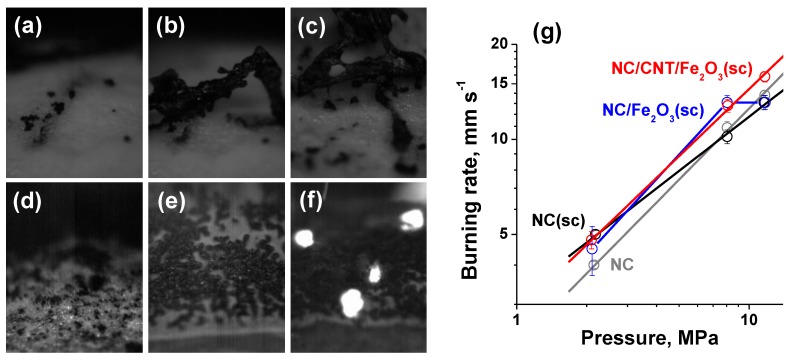
Combustion of the composites: still images from the high-speed filming (**a–f**) and the burning rate-pressure dependencies (**g**). Images shows the typical shots of NC (**a**,**b**), *NC(sc)* (**c**), *NC/CNT(sc)* (**d**), *NC/Fe_2_O_3_(sc)* (**e**), and *NC/CNT/Fe_2_O_3_(sc)* (**f**) burning. Frame width is 1.22 mm.

The energetic performance of the samples was estimated by combustion in air with the high-speed filming of the burning surface (2000 fps) and directly measured as the burning rate–pressure dependency in a Crawford bomb at elevated pressures. For the raw nitrocellulose, the observation revealed active bubble formation on the surface during combustion (Videos S1,S2, Supporting information). In this foam zone, small black particles were formed that moved inside the liquid layer to aggregate (see Figure 5a,b for sequential frames with 0.075 s time difference). Thus, the formed framework remained above the surface until burning out in a flame of the final reactions between the semiproducts of NC decomposition or until blowing away by the gas flow. Similar observations were drawn for the *NC(sc)* sample with the only exception of higher carbonaceous framework formation that remained after tests as the solid residue (Figure 5c and Figure S6, Video S2, Supporting information). The finer structure of unreacted nano-nitrocellulose seemed to either result in a more distributed localization of the seeds or in the structural modification of the formed framework to keep it intact in gas flow. Considering the burning rate values, the nano-NC exhibited a higher velocity as compared to raw NC, e.g., 3.8 against 4.7 mm·s^−1^ at 2 MPa (Figure 5g). This enhancement was apparently linked with the lowering of the activation barrier for condensed-phase decomposition as revealed by thermal analysis. The introduction of carbon nanotubes gave more seeds for carbonaceous framework (Figure 5d), and in fact a considerable porous layer grew above the surface (Figure S7, Video S3, Supporting information). However, it easily broke down into fragments, which were blown away as the combustion proceeds. The burning rate for the *NC/CNT(sc)* composite was the same as for nano-NC monopropellant showing no significant effect of the increased heat conductivity on the combustion front propagation.

Figure 5e illustrates the burning process for the NC nanocomposite modified with nano-Fe_2_O_3_ addition. Seeds of the size bigger than that in previous samples stuck together on the surface and stayed in gas phase as a skeleton (Video S4, Supporting information). Recently, Shin et al. have proposed a facile method of nano-Fe_2_O_3_ transformation to Fe_3_O_4_ nanoparticles covered by a carbonaceous layer using a porous film made of nano-Fe_2_O_3_ particles filled with nitrocellulose [52]. After the burning of the NC matrix with the registered temperatures of 500–900 °C, the desired product was recovered. In line with these results, Denisyuk and Demidova [24] noticed the reduction of SnO_2_ to a mixture of SnO and Sn during the combustion of an NC-based propellant. Here, we could assume the reduction of nano-Fe_2_O_3_ in our experiments; however the necessary high-temperature flame was not registered. As for the burning rate, we noticed the acceleration of the combustion by iron oxide addition under 8 MPa pressure (blue curve, Figure 5g). 

When carbon nanotubes and nano-iron oxide are introduced together to the composite, we observe a different picture of combustion (Video S5, Supporting information). Figure 5f shows a typical shot of the burning surface covered by a crumbly carbonaceous layer with luminous spots that appeared occasionally. These luminous regions give strong evidence of the catalyzed reactions and the increased heat flux seems to be the main driver for the combustion velocity increase. Figure 5g summarizes the burning rate-pressure data showing the highest catalytic effect at high pressures, achieving a maximum increase in 20% at 12 MPa (Table 1). The simplified combustion models [53,54] assumed the pressure exponent *n* of the burning rate dependency *U* = *B*·*P*^n^ to be an indicator of the importance of gas-phase reactions, and the observed increase in it is consistent with the action of the catalyst in gas-phase zone. 

To estimate the value of the SAS process in the arrangement of nanocatalyst and CNTs, we prepare the sample of the same composition *NC/CNT/Fe_2_O_3_* by means of conventional dry mixing. Testing for sensitivity and combustion reveal its inferior performance as compared to the composite produced via the SAS (Table 1). 

## 4. Conclusions

Supercritical CO_2_ has been applied for the first time as anti-solvent for obtaining nano-sized nitrocellulose powder and various nitrocellulose-based composites. Thus prepared materials exhibit a significantly lower sensitivity to friction and a higher burning rate than the raw fibrous nitrocellulose, while maintaining the chemical composition and thermal stability. For the benchmark dry mixture of *NC/CNT/Fe_2_O_3_* the results are notably lower than those for the SAS-produced composite. Overall, the proposed approach for introducing the burning rate modifiers into the matrices of energetic materials allows increasing the performance, satisfies the high technological and ecological requirements and may be useful for modern energetic composites production.

## Figures and Tables

**Table 1:** Summary of properties for raw NC and SAS-obtained composites.

Sample	Decomposition onset ^1^, °C	Heat Effect ^1^, J·g^−1^	Friction Sensitivity, N	Burning Rate ^2^, mm·s^−1^
*NC*	193	1880 ± 90	234 ± 21	13.8 ± 0.5
*NC(sc)*	189	1970 ± 30	342 ± 18	13.1 ± 0.5
*NC/CNT(sc)*	188	1920 ± 150	>360 (10%)	13.2 ± 0.5
*NC/Fe_2_O_3_(sc)*	186	1790 ± 150	229 ± 18	13.1 ± 0.5
*NC/CNT/Fe_2_O_3_(sc)*	189	1890 ± 150	>360 (40%)	15.8 ± 0.6
*NC/CNT/Fe_2_O_3_*	193	1590 ± 150	222 ± 31	14.6 ± 0.5

^1^ Measured at 5 K·min^−1^ rate. ^2^ For pressed pellets burned at 12 MPa.

## Supplementary Materials

The following are available online at https://www.mdpi.com/2079-4991/9/10/1386/s1: Section S1: Experimental Methods, Section S2: Starting Materials, Figure S1: Microstructure of (a, b) untreated micron-sized NC fibers, (c,d) carbon nanotubes, (e,f) nano-sized iron oxide, Section S3: Details of the SAS process, Table S1: SAS process parameters for NC modification, Section S4: Morphology of Nano-NC, Figure S2: Atomic force microscopy (AFM) image of nitrocellulose powder produced via SAS process, Table S2: Parameters of the particle-size distribution of nano-NC powder, Section S5: Determination of catalyst content in fabricated composites, Figure S3: Temperature and mass profiles during annealing of NC-based composites with Fe_2_O_3_ addition, Section S6: Thermokinetic analysis, Figure S4: Results of formal kinetic analysis for micron-(a) and nano-(b) sized nitrocellulose, Figure S5: Data from review and the results of the current study, Section S7: Combustion tests, Figure S6: Combustion of untreated (a–c) and processed (d–f) nitrocellulose: (a,d) view of pellet before test, (b,e) still image of the burning surface, (c,f) residue after experiment, Figure S7: Combustion nitrocellulose with additives of nano-iron oxide (a–c), carbon nanotubes (d–f) and both Fe_2_O_3_ and CNTs (g–i): (a,d,g) view of pellet before test, (b,e,h) still image of the burning surface, (c,f,i) residue after experiment, Table S3: Results of combustion experiments, Figure S8: Combustion velocity against pressure for all involved compositions, Videos S1–S6.

## Nomenclature

A	Pre-exponential factor
ABPR	Automated back pressure regulator
B	Burn rate coefficient
CNTs	Carbon nanotubes
*E*_a_	Apparent activation energy
EtOH	Ethanol
f	Feeding rate of NC-solution/suspension
F	Feeding rate of CO_2_
MBPR	Manual back pressure regulator
MeOH	Methanol
micron-NC	Nitrocellulose microfibers (raw nitrocellulose)
n	Pressure exponent
nano-NC	Nitrocellulose nanoparticles
NC	Nitrocellulose
NC(sc)	Nitrocellulose particles obtained by SAS process
NC/CNT(sc)	Nitrocellulose composite with CNTs obtained by SAS process
NC/Fe_2_O_3_(sc)	Nitrocellulose with nano-Fe_2_O_3_ obtained by SAS process
NC/CNT/Fe_2_O_3_(sc)	Nitrocellulose with CNTs and nano-Fe_2_O_3_ obtained by SAS process
P	Pressure
*P*_c_	Critical pressure of CO_2_
RDX	1,3,5-trinitro-1,3,5-triazinane
SAS	Supercritical Anti-Solvent process
sc-CO_2_	Supercritical carbon dioxide
SEM	Scanning electron microscopy
*T*_c_	Critical temperature of CO_2_
TEM	Transmission electron microscopy
U	Burning rate
