# Supplementary material for: Supercritical Antisolvent Processing of Nitrocellulose: Downscaling to Nanosize, Reducing Friction Sensitivity and Introducing Burning Rate Catalyst
Source: Nanomaterials (Basel). 2019 Sep 27;9(10):1386. doi: 10.3390/nano9101386 (PMC6835986; doi:10.3390/nano9101386)
Supplement: Supplementary file 1 [file nanomaterials-09-01386-s001.pdf]

**Supporting Information**

# Supercritical Antisolvent Processing of Nitrocellulose: Downscaling to Nanosize, Reducing Friction Sensitivity and Introducing Burning Rate Catalyst

**Oleg S. Dobrynin [1], Mikhail N. Zharkov [1], Ilya V. Kuchurov [1], Igor V. Fomenkov [1], Sergey G. Zlotin [1], Konstantin A. Monogarov [2], Dmitry B. Meerov [2], Alla N. Pivkina [2] and Nikita V. Muravyev [2,*]**

[1] N.D. Zelinsky Institute of Organic Chemistry, Russian Academy of Sciences; Moscow119991, Russia; oleg_dobrynin@list.ru (O.S.D.); m.n.zharkov@gmail.com (M.N.Z.); kuchurov@mail.ru (I.V.K.); ootx@ineos.ac.ru (I.V.F.); zlotin@ioc.ac.ru (S.G.Z.)

[2] N.N. Semenov Federal Research Center for Chemical Physics, Russian Academy of Sciences; Moscow 119991, Russia; k.monogarov@gmail.com (K.A.M.), mmeerov@mail.ru (D.B.M.), alla_pivkina@mail.ru (A.N.P.)

* Correspondence: n.v.muravyev@ya.ru; Tel.: +7-499-137-8203

**Contents**

Nomenclature ... 2
Section S1. Experimental Methods ... 3
Section S2. Starting Materials ... 4
Section S3. Details of the SAS process ... 6
Section S4. Morphology of Nano-NC ... 8
Section S5. Determination of catalyst content in fabricated composites ... 9
Section S5. Determination of catalyst content in fabricated composites ... 9
Section S6. Thermokinetic analysis ... 10
Section S7. Combustion tests ... 12
Section S8. References ... 16

**Nomenclature**

| | |
|---|---|
| $A$ | Pre-exponential factor |
| ABPR | Automated back pressure regulator |
| $B$ | Burn rate coefficient |
| CNTs | Carbon nanotubes |
| $E_a$ | Apparent activation energy |
| EtOH | Ethanol |
| $f$ | Feeding rate of NC-solution/suspension |
| $F$ | Feeding rate of $CO_2$ |
| MBPR | Manual back pressure regulator |
| MeOH | Methanol |
| micron-NC | Nitrocellulose microfibers (raw nitrocellulose) |
| $n$ | Pressure exponent |
| nano-NC | Nitrocellulose nanoparticles |
| NC | Nitrocellulose |
| *NC(sc)* | Nitrocellulose particles obtained by SAS process |
| *NC/CNT(sc)* | Nitrocellulose composite with CNTs obtained by SAS process |
| *NC/*$Fe_2O_3$*(sc)* | Nitrocellulose with nano-$Fe_2O_3$ obtained by SAS process |
| *NC/CNT/*$Fe_2O_3$*(sc)* | Nitrocellulose with CNTs and nano-$Fe_2O_3$ obtained by SAS process |
| $P$ | Pressure |
| $P_c$ | Critical pressure of $CO_2$ |
| RDX | 1,3,5-trinitro-1,3,5-triazinane |
| SAS | Supercritical Anti-Solvent process |
| sc-$CO_2$ | Supercritical carbon dioxide |
| SEM | Scanning electron microscopy |
| $T_c$ | Critical temperature of $CO_2$ |
| TEM | Transmission electron microscopy |
| $U$ | Burning rate |

**Section S1. Experimental Methods**

*Electron microscopy.* Target-oriented approach was utilized for the optimization of the analytic measurements [1]. Before measurements the samples were mounted on a 25 mm aluminum specimen stub and fixed by conductive adhesive. Metal coating with a thin film (10 nm) of platinum/palladium alloy (80/20) was performed using magnetron sputtering method as described earlier [2]. The observations were carried out using Hitachi SU8000 field-emission scanning electron microscope (FE-SEM). Images were acquired in secondary electron mode at 2 kV accelerating voltage. Morphology of the samples was studied taking into account possible influence of metal coating on the surface [2].

Samples morphology was studied also using Hitachi transmission electron microscope (TEM). Before measurements the samples were mounted on a 3 mm copper grid and fixed in a grid holder. Images were acquired in bright-field TEM mode at 100 kV accelerating voltage.

*Atomic force microscopy.* Morphology of the samples was revealed by scanning probe microscopy (NTEGRA Prima microscope, NT-MDT) with ≤ 10 nm and ~1nm cantilevers. Powdered samples were pressed in small pellets and its surface was monitored in semi-contact or contact scanning modes.

*Specific surface area.* The BET surface area was determined using FlowSorb III 2305 (Micromeritics) instrument by measuring the adsorption of a gas mixture (30%$N_2$/70%He) on the surface of powder.

*Elemental analysis* was revealed by Perkin Elmer 2400 Series analyzer.

*Thermal analysis.* Thermogravimetric (TGA) runs were performed with Netzsch STA 449 F3 thermal analyzer. Samples ca. 1 mg weight have been poured in alumina pans, covered with pierced lids, and linearly heated at 5 K $min^{-1}$ rate in argon flow. Differential scanning calorimetry (DSC) was registered in separate experiments in Netzsch DSC 204 HP apparatus. Nearly 0.5 mg of samples were placed in aluminum crucibles, covered with pierced lids, and linearly heated with the same rate of 5 K $min^{-1}$ in nitrogen flow.

*Friction sensitivity* was determined in full set of experiments according to STANAG 4487 [3].

**Section S2. Starting Materials**

Scanning electron and probe microscopy have been used to disclose the morphology of the involved powders. Figures S1a, b represent the view of as-received nitrocellulose fibers showing a smooth surface with some cracks. Carbon nanotubes appeared as the elongated tortuous objects. In line with the manufacturer specification [4] the inner diameter of CNTs is ca.10 nm, the outer diameter is near 20 nm, and the length is no less than 2 μm (Figures S1c, b). Iron oxide is formed mainly by nanoparticles as evidenced from both types of microscopy (Figure S1e, f). The specific surface area for nano-$Fe_2O_3$ after degassing the powder at 120 °C is 12.8 ± 0.3 $m^2$ $g^{-1}$, higher thermosetting (300 °C) reveals the larger value of 14.3 ± 0.3 $m^2$ $g^{-1}$. High purity carbon dioxide (Russian standard TU 2114-006-05015259-2014) and acetone (Russian standard TU 2633-012-29483781-2009) were used for synthesis.

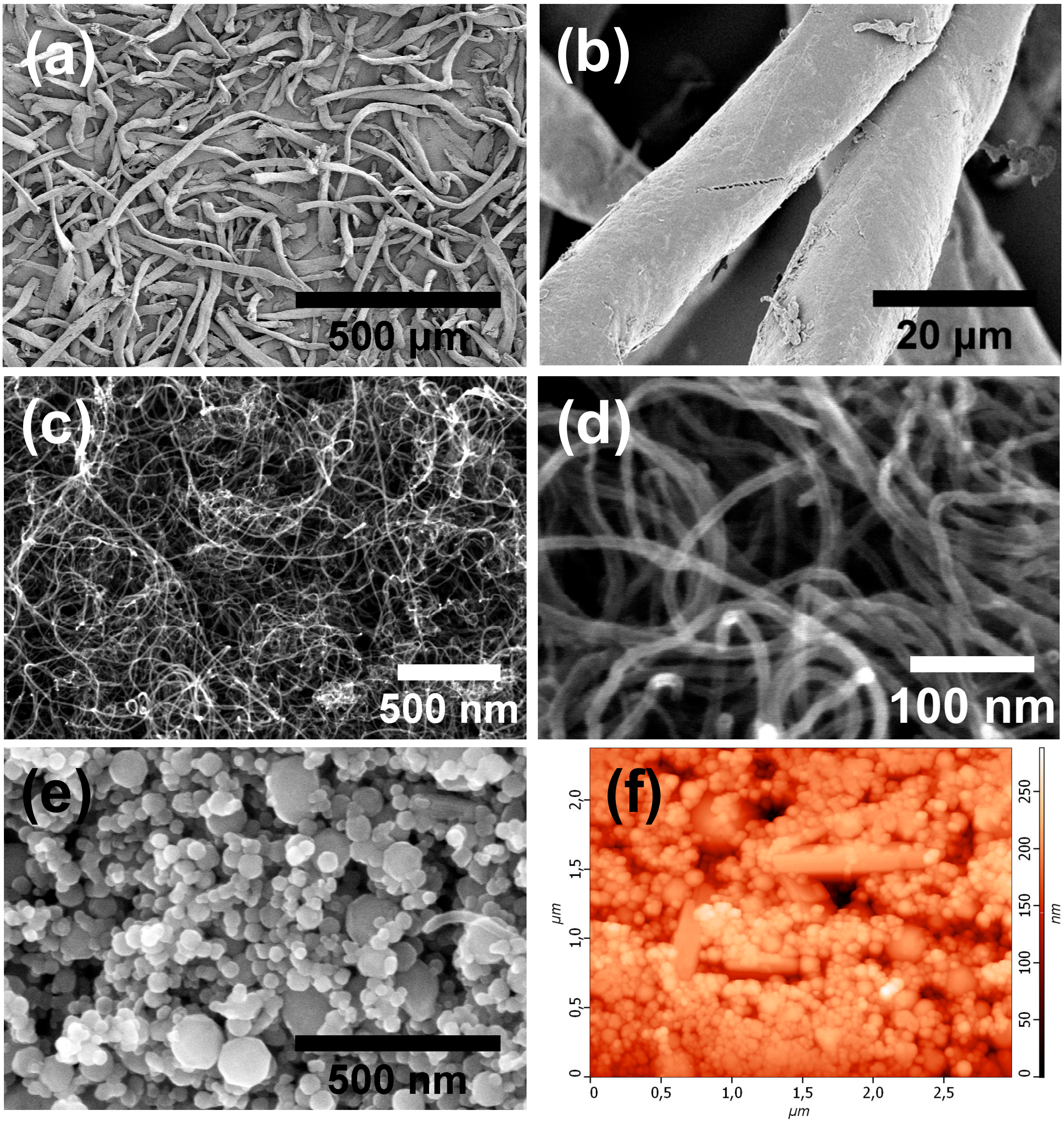


**Figure S1.** Microstructure of (a, b) untreated micron-sized NC fibers, (c, d) carbon nanotubes, (e, f) nano-sized iron oxide.

**Section S3. Details of the SAS process**

Table S1 summarizes the performed experiments with identification of the following governing parameters: the nozzle diameter *D*, autoclave pressure *P*, the ratio between solution (suspension) and $CO_2$ feeding rates (*f*/*F*), the volume of solvent *V*, the NC mass *m*(NC) and its concentration in solution (suspension), *w*(NC), the additive type present, and the characteristics of the final product – its morphology and yield (*N* stands for number of runs).

**Table S1.** SAS process parameters for NC modification

| expt # | *D*, μm | *P*, bar | *f*/*F*, $cm^3/g$ | *V*, ml | *m*(NC), g | *w*(NC), wt.% | CNT | $Fe_2O_3$ | Product appearance | Yield, % | *N* |
|---|---|---|---|---|---|---|---|---|---|---|---|
| 1 | 150 | 100 | 1/50 | 20 | 0.159 | 1 | - | - | uniform powder | 81 | 1 |
| 2 | 150 | 110 | 1/50 | 20 | 0.158 | 1 | - | - | gel-like recovered product | 39 | 2 |
| 3 | 150 | 90 | 1/50 | 20 | 0.158 | 1 | - | - | gel-like recovered product | 11 | 1 |
| 4 | 150 | 120 | 1/50 | 20 | 0.158 | 1 | - | - | gel-like recovered product | 13 | 1 |
| 6 | 150 | 100 | 2/50 | 40 | 0.316 | 1 | - | - | gel-like recovered product | 27 | 1 |
| 7 | 150 | 100 | 1/50 | 31 | 0.488 | 2 | - | - | gel-like recovered product | 59 | 1 |
| 8 | 150 | 100 | 1/50 | 20 | 0.488 | 3 | - | - | gel-like recovered product | 39 | 1 |
| 9 | 200 | 100 | 2/50 | 30 | 0.288 | 1 | - | - | gel-like recovered product | 43 | 1 |
| 10 | 100 | 100 | 4/50 | 30 | 0.288 | 1 | - | - | gel-like recovered product | 45 | 1 |
| 11 | 760 | 90 | 1/50 | 40 | 0.675 | 2 | - | - | "herring-bone" structures and uniform powder on filter | 83 | 1 |
| 12 | 760 | 90 | 2/50 | 61 | 1.084 | 2 | - | - | "herring-bone" structures and uniform powder on filter | 85 | 1 |
| 13 | 760 | 90 | 4/50 | 61 | 1.087 | 2 | - | - | "herring-bone" structures and uniform powder on filter | 82 | 1 |
| 14 | 760 | 100 | 4/50 | 61 | 1.067 | 2 | - | - | "herring-bone" structures and uniform powder on filter | 83 | 1 |
| 15 | 760 | 120 | 1/50 | 61 | 1.024 | 2 | - | - | uniform powder | 88 | 1 |
| 16 | 760 | 120 | 4/50 | 61 | 1.012 | 2 | - | - | uniform powder | 88 | 1 |
| 17 | 760 | 100 | 2/50 | 126 | 1.004 | 1 | - | - | "herring-bone" structures and uniform powder on filter | 92 | 1 |
| 18 | 760 | 100 | 2/50 | 41 | 1.023 | 3 | - | - | uniform powder | 87 | 1 |

| | | | | | | | | | | | |
|---|---|---|---|---|---|---|---|---|---|---|---|
| 19 | 150 | 120 | 2/50 | 30 | 0.5 | 2 | - | - | gel-like recovered product | - | 1 |
| 20 | 760 | 100 | 1/50 | 63 | 1 | 2 | - | - | uniform powder | 88 | 2 |
| 21 | 760 | 100 | 1/50 | 61 | 1.08 | 2 | + | - | uniform powder | 88 | 2 |
| 22 | 760 | 100 | 1/50 | 40 | 0.634 | 2 | + | + | uniform powder | 87 | 1 |
| 23 | 760 | 100 | 2/50 | 61 | 1 | 2 | - | - | uniform powder | 86 | 1 |
| 24 | 760 | 150 | 1/50 | 40 | 0.668 | 2 | - | - | uniform powder | 96 | 1 |
| 25 | 760 | 120 | 2/50 | 60 | 1 | 2 | - | - | uniform powder | 91 | 20 |
| 26 | 760 | 120 | 2/50 | 61 | 1.06 | 2 | - | + | uniform powder | 91 | 2 |
| 27 | 760 | 100 | 2/50 | 61 | 1.024 | 2 | + | - | uniform powder | 87 | 3 |
| 28 | 760 | 100 | 2/50 | 61 | 1.019 | 2 | + | + | uniform powder | 93 | 2 |
| 29 | 760 | 100 | 2/50 | 61 | 1.011 | 2 | - | + | uniform powder | 89 | 2 |
| 30 | 760 | 120 | 2/50 | 61 | 1.097 | 2 | - | - | uniform powder | 86 | 1 |

Nickel and copper salicylates were intended to be introduced same as the iron oxide since they are known as catalysts for NC-based propellants [5]. But both salts appeared to be soluble in acetone-$CO_2$ binary system, thus not suitable for the selected process.

**Section S4. Morphology of Nano-NC**

Particle size distribution for nano-NC powder has been obtained by counting particles in AFM images. Typical AFM image is presented in Figure S2 and details of the numerical particle-size distribution can be found in Table S2.

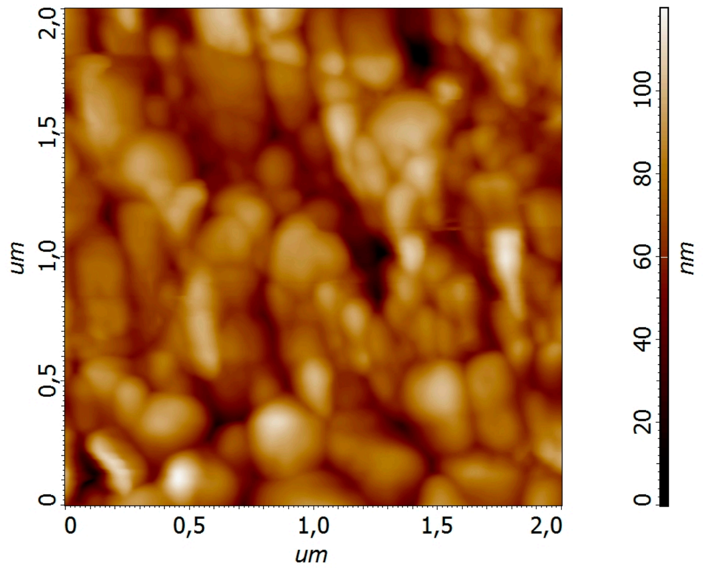


**Figure S2.** AFM image of nitrocellulose powder produced via SAS process.

**Table S2.** Parameters of the particle-size distribution of nano-NC powder

| | |
|---|---|
| Number of particles | 216 |
| Average diameters | |
| <D>, μm | 194 |
| D43, μm | 276 |
| Cummulative distribution | |
| D10, μm | 80 |
| D50, μm | 180 |
| D90, μm | 290 |
| Log-normal approximation | |
| Xc, μm | 195 |
| σ | 0.46 |

**Section S5. Determination of catalyst content in fabricated composites**

Considering the non-100% recovery of the product for NC composites with additive (see Table S1), the exact additive content in material can differ from that in original suspension, i.e., 1 wt.% of CNTs and 5 wt.% of nano-$Fe_2O_3$ above the 100% of NC. To check if this content remains the same in the final product, we perform the gentle annealing of the composites to receive the iron oxide residue. Figure S3 illustrates the heating program, the key issue is to use the low heating rates and sample masses during NC-core decomposition to prevent ignition of the sample and low recovery of the residue (e.g., 0.5 wt.% after 10 K/min heating to 300°C in preliminary tests). Sample loads were near 10 mg, low enough to eliminate self-heating, and appropriate to give a sufficient accuracy of weighting the residue.

We start with mechanical mixture of NC/CNT/$Fe_2O_3$ where there should be no loss of material during preparation. Brown residue after tests is 5.5 ± 0.2 wt.% of the initial mass, close to target 5/(100 + 1 + 5)·100 = 4.7% considering the sampling inhomogeneity, weighting errors, and especially the not-complete annealing of the carbonaceous residues. For composites prepared with SAS process, the catalyst content was 5.3 ± 0.5 % for NC/$Fe_2O_3$(sc) and 5.0 ± 0.3 % for NC/CNT/$Fe_2O_3$(sc).

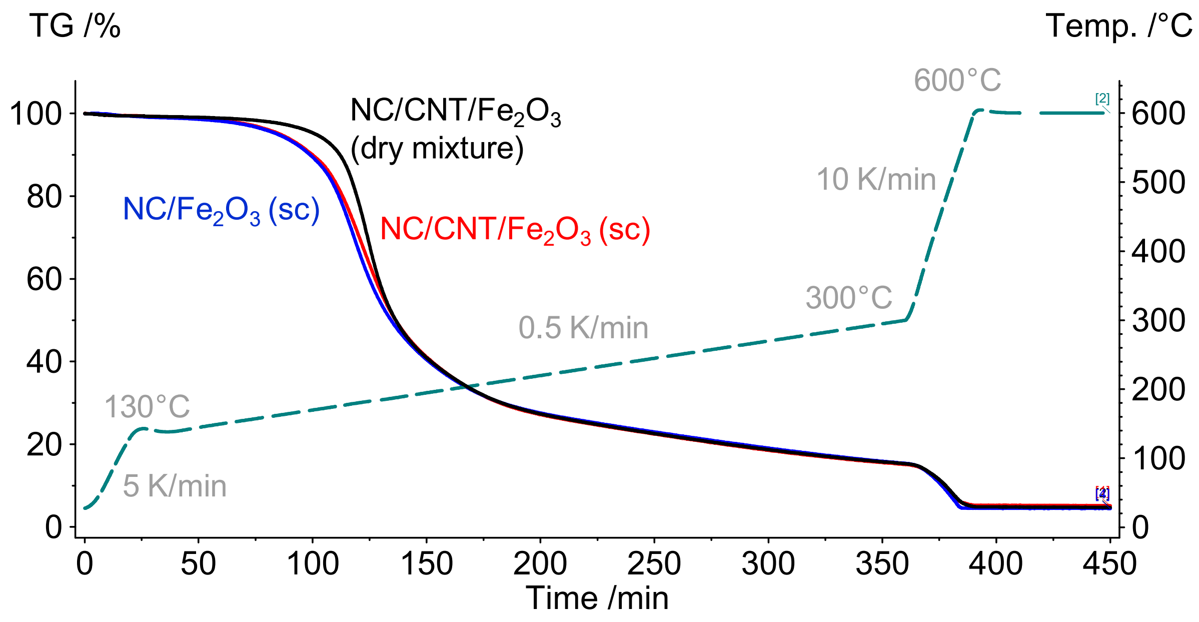


**Figure S3.** Temperature and mass profiles during annealing of NC-based composites with $Fe_2O_3$ addition.

## Section S6. Thermokinetic analysis

Figure S4 shows the results of formal kinetic analysis of DSC curves obtained at 0.5-5 K/min for micron-(a) and nano-(b) NC. Non-linear regression estimates of the kinetic parameters and information criteria showing that single-step kinetic models outperform the two-step models are presented in figures.

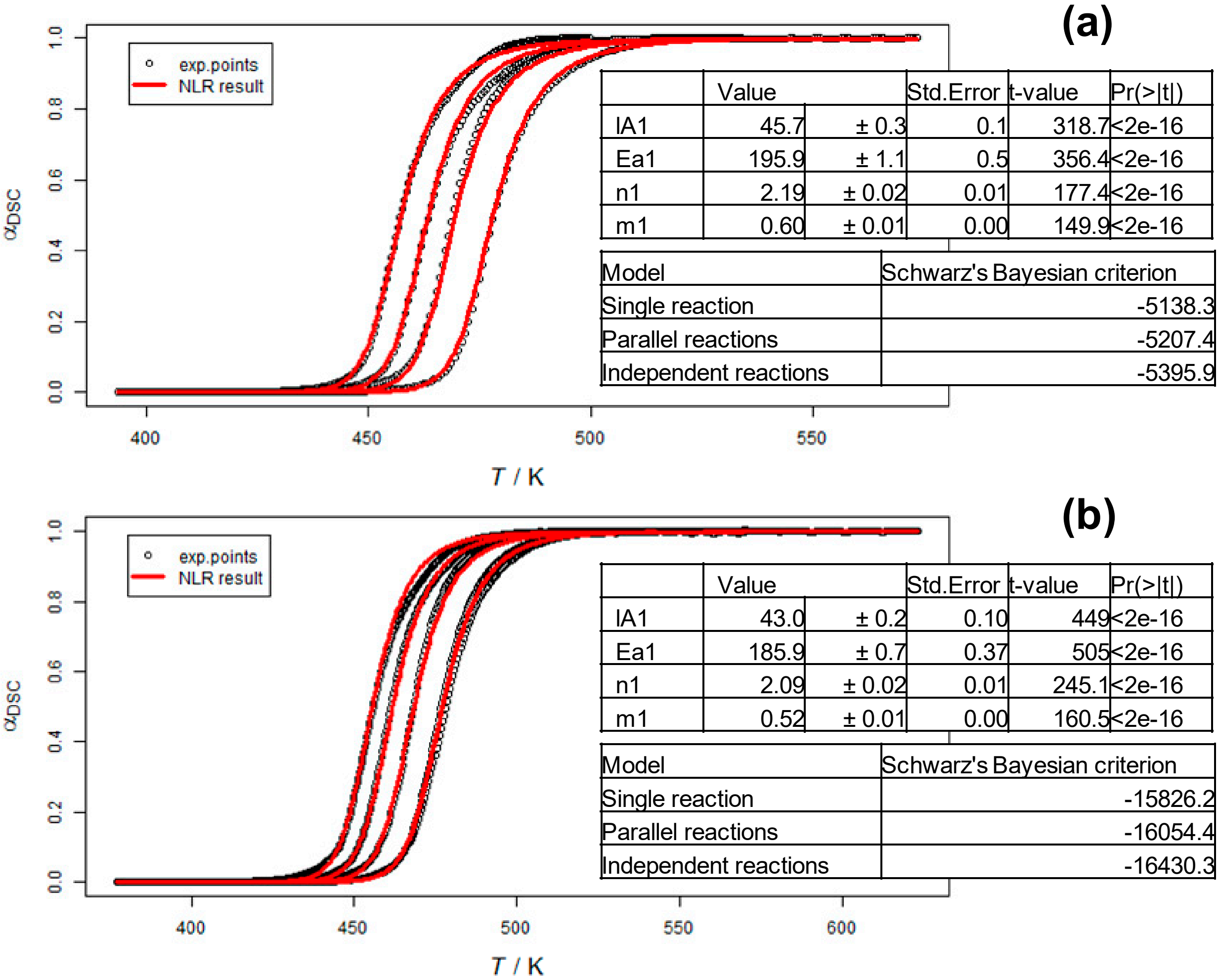


(a)

| | Value | | Std.Error | t-value | Pr(>\|t\|) |
|---|---|---|---|---|---|
| lA1 | 45.7 | ± 0.3 | 0.1 | 318.7 | <2e-16 |
| Ea1 | 195.9 | ± 1.1 | 0.5 | 356.4 | <2e-16 |
| n1 | 2.19 | ± 0.02 | 0.01 | 177.4 | <2e-16 |
| m1 | 0.60 | ± 0.01 | 0.00 | 149.9 | <2e-16 |

| Model | Schwarz's Bayesian criterion |
|---|---|
| Single reaction | -5138.3 |
| Parallel reactions | -5207.4 |
| Independent reactions | -5395.9 |

(b)

| | Value | | Std.Error | t-value | Pr(>\|t\|) |
|---|---|---|---|---|---|
| lA1 | 43.0 | ± 0.2 | 0.10 | 449 | <2e-16 |
| Ea1 | 185.9 | ± 0.7 | 0.37 | 505 | <2e-16 |
| n1 | 2.09 | ± 0.02 | 0.01 | 245.1 | <2e-16 |
| m1 | 0.52 | ± 0.01 | 0.00 | 160.5 | <2e-16 |

| Model | Schwarz's Bayesian criterion |
|---|---|
| Single reaction | -15826.2 |
| Parallel reactions | -16054.4 |
| Independent reactions | -16430.3 |

**Figure S4.** Results of formal kinetic analysis for micron- (a) and nano-(b) sized nitrocellulose. Computations performed in THINKS thermokinetic software [6].

The obtained kinetic parameters were compared with the extensive data from review by Brill and Gongwer [7]. Figure S5 shows that our results fall to the kinetic compensation line that is supposed to include the autocatalytic reaction parameters.

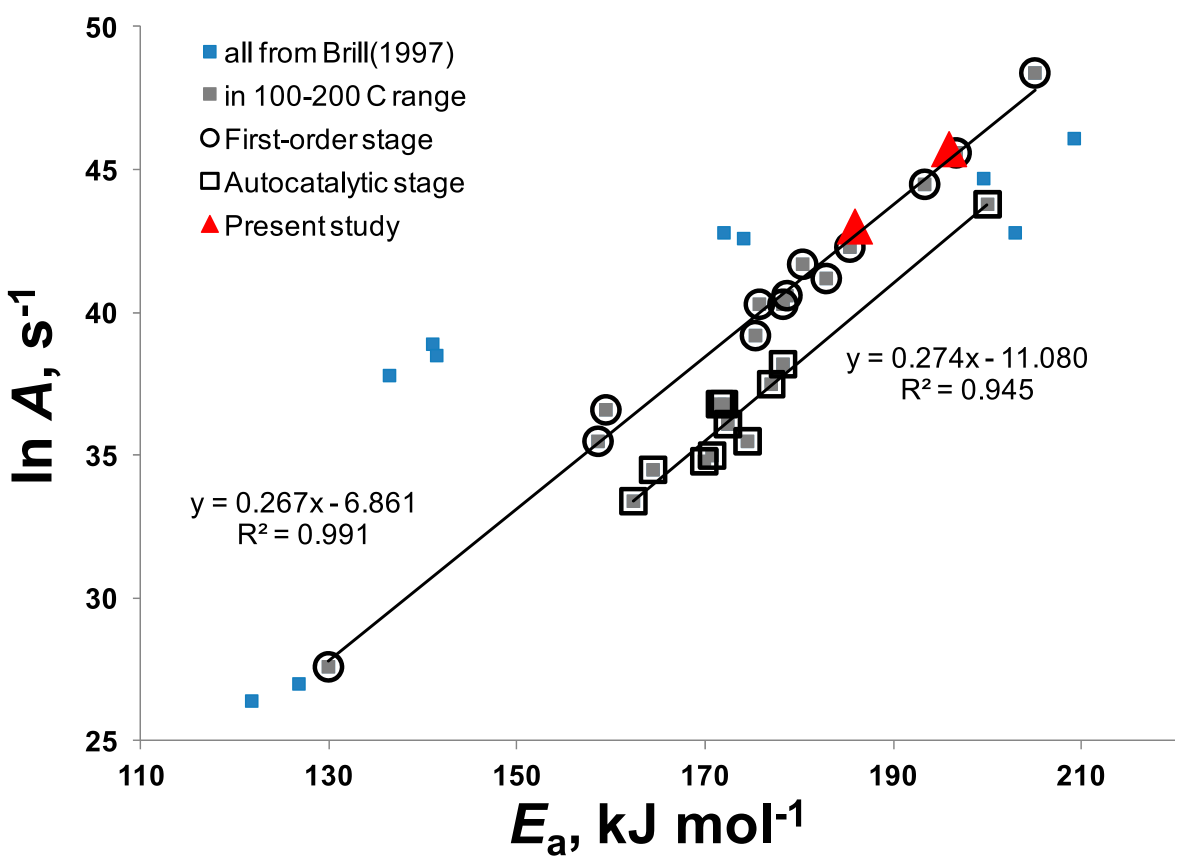


**Figure S5.** Data from review [7] and the results of the current study.

**Section S7. Combustion tests**

Figure S6 shows the images of pressed nitrocellulose samples before and after experiment, as well as a frame from video during combustion. For nano-sized NC the amount of residue is notably higher that for micro-sized sample.

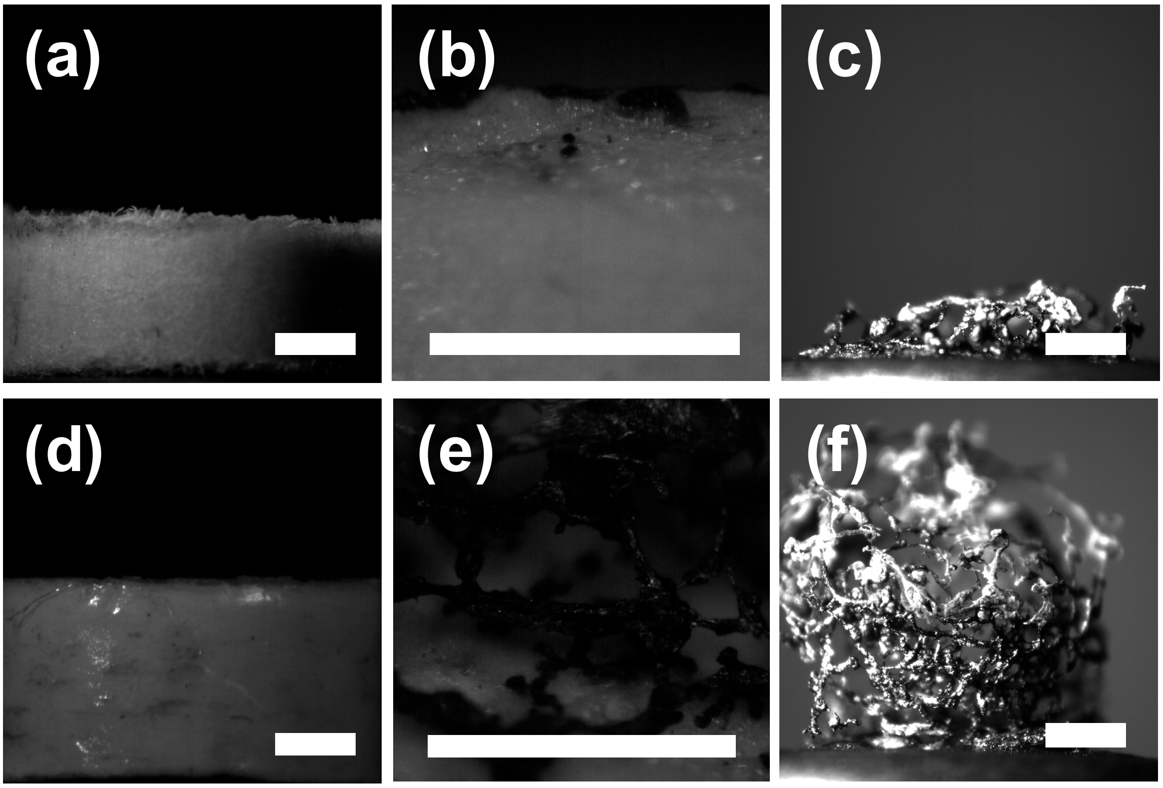


**Figure S6.** Combustion of untreated (a-c) and processed (d-f) nitrocellulose: (a, d) view of pellet before test, (b, e) still image of the burning surface, (c, f) residue after experiment. Length of white bar is 1 mm.

Analogously, the views of the samples before, during, and after combustion are presented in Figure S7. The additive type apparently influences the appearance of the combustion surface, the view and amount of the combustion residue.

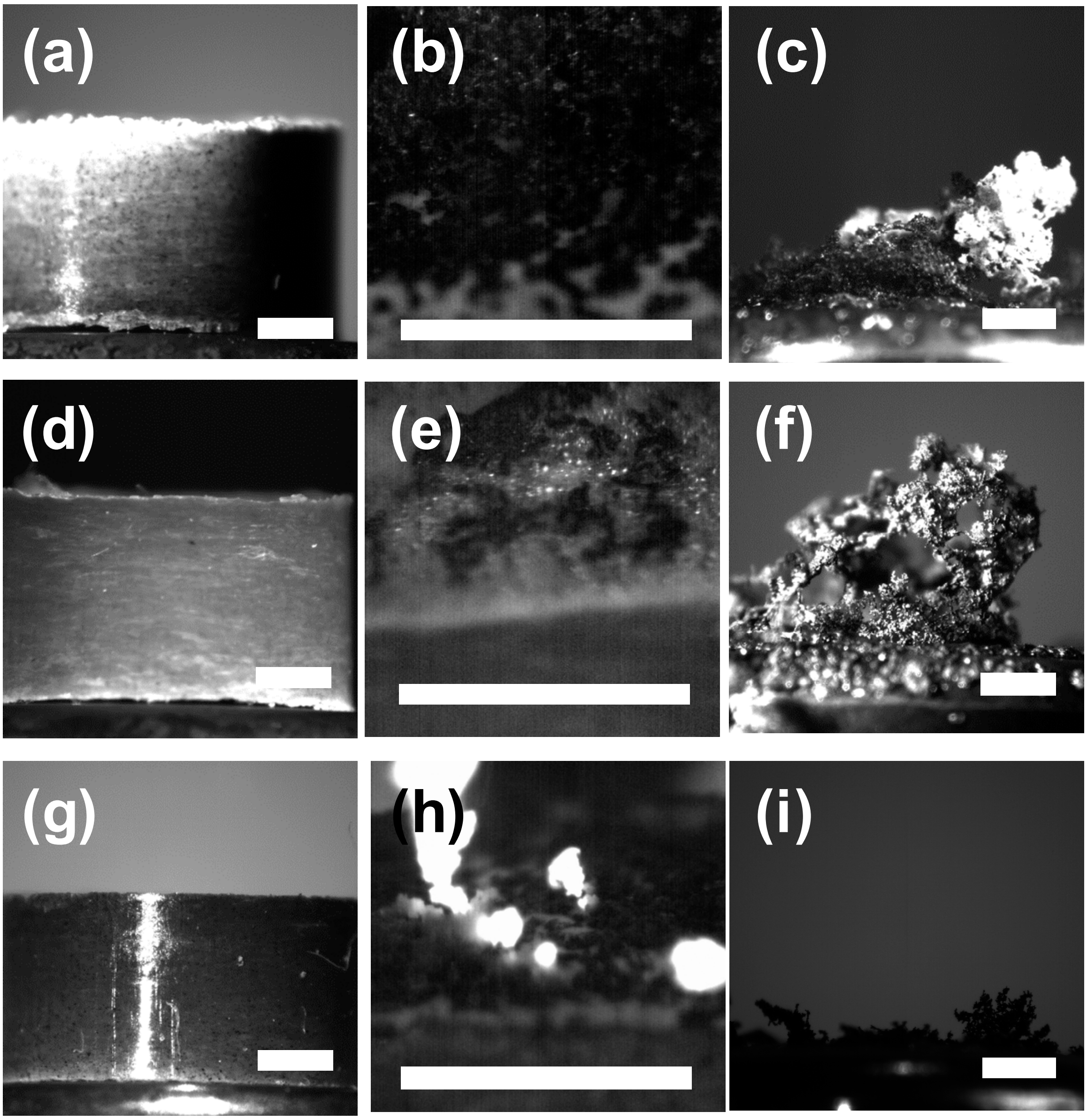


**Figure S7.** Combustion nitrocellulose with additives of nano-iron oxide (a-c), carbon nanotubes (d-f) and both $Fe_2O_3$ and CNTs (g-i) : (a, d, g) view of pellet before test, (b, e, h) still image of the burning surface, (c, f, i) residue after experiment. Length of white bar is 1 mm.

Samples have been pressed at 300 MPa in cylinders with 6 mm diameter. Charges of two pellets (~0.5 g) were coated on the lateral surface and put in Crawford bomb. Combustion at 2 MPa of nitrogen was monitored by pressure rise and video-registration (at 1200 fps). Filming of the burning surface (2000 fps) was performed on uncoated 5 mm pellets in open air.

**Table S3.** Results of combustion experiments

| # | Sample | Density, g $cm^{-3}$ | Porosity, % | Burning rate, mm $s^{-1}$at $P$ [MPa] | | | Parameters in $U = B·P^v$ | | |
|---|---|---|---|---|---|---|---|---|---|
| | | | | 2 | 8 | 12 | $B$ | $v$ | $R^2$ |
| 1 | NC | 1.41 ± 0.01 | 15 | 4.0 ± 0.1 | 10.9 ± 0.5 | 13.8 ± 0.5 | 2.24 ± 0.08 | 0.75 ± 0.02 | 0.998 |
| 2 | NC(sc) | 1.40 ± 0.05 | 15 | 5.0 ± 0.1 | 10.2 ± 0.5 | 13.1 ± 0.5 | 3.17 ± 0.11 | 0.57 ± 0.02 | 0.998 |
| 3 | NC/CNT(sc) | 1.42 ± 0.02 | 15 | 5.0 ± 0.3 | 10.6 ± 0.5 | 13.2 ± 0.5 | 3.2 ± 0.2 | 0.57 ± 0.03 | 0.996 |
| 4 | NC/$Fe_2O_3$(sc) | 1.44 ± 0.05 | 13 | 4.5 ± 0.8 | 13.1 ± 0.5 | 13.1 ± 0.5 | –[a] | –[a] | –[a] |
| 5 | NC/CNT/$Fe_2O_3$(sc) | 1.44 ± 0.05 | 13 | 4.8 ± 0.3 | 12.8 ± 0.6 | 15.8 ± 0.6 | 2.8 ± 0.2 | 0.71 ± 0.03 | 0.995 |
| 6[b] | NC/CNT/$Fe_2O_3$ | 1.43 ± 0.01 | 14 | 5.3 ± 0.3 | 11.8 ± 0.6 | 14.6 ± 0.6 | 3.4 ± 0.2 | 0.60 ± 0.03 | 0.999 |

[a] Values cannot be fitted by a single power dependency.

[b] Composition #6 was prepared by dry mixing of the as-received NC, iron oxide and CNTs. All attempts to mix in liquid with ultrasonic vibrations or mechanical agitation result in films that even when cut in small pieces reveal the serious cracking in pressed pellets.

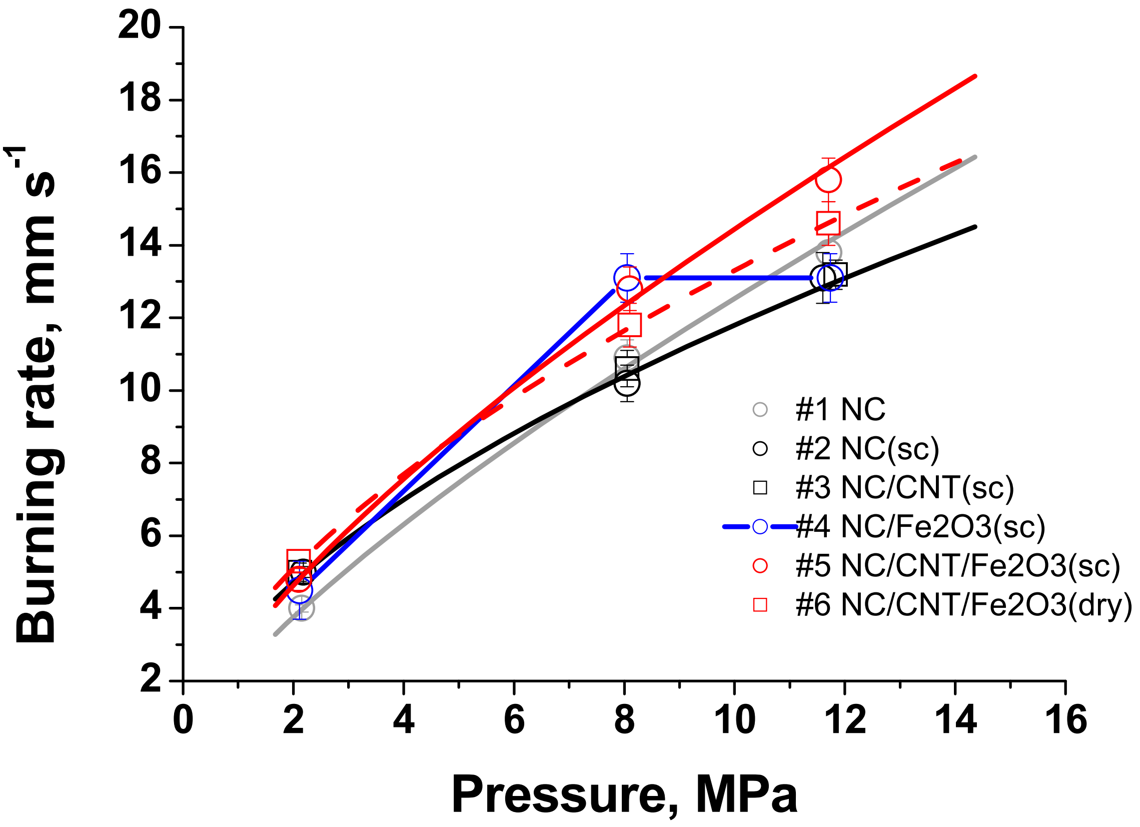


**Figure S8.** Combustion velocity against pressure for all involved compositions.

**Video 1 is available at https://youtu.be/UwP2gr4oHuo**

**Video 2 is available at https://youtu.be/rs3KKf5Uk6w**

**Video 3 is available at https://youtu.be/UE8-GHeiIWM**

**Video 4 is available at https://youtu.be/VcvbKoOyNv8**

**Video 5 is available at https://youtu.be/MmoUUPHsFOo**

**Video 6 is available at https://youtu.be/xeesBCVG_MU**

**Section S8. References**


[1] V.V. Kachala, L.L. Khemchyan, A.S. Kashin, N.V. Orlov, A.A. Grachev, S.S. Zalesskiy, V.P. Ananikov, Target-oriented analysis of gaseous, liquid and solid chemical systems by mass spectrometry, nuclear magnetic resonance spectroscopy and electron microscopy, Russian Chemical Reviews. 82 (2013) 648–685. doi:10.1070/RC2013v082n07ABEH004413.

[2] A.S. Kashin, V.P. Ananikov, A SEM study of nanosized metal films and metal nanoparticles obtained by magnetron sputtering, Russian Chemical Bulletin. 60 (2011) 2602–2607. doi:10.1007/s11172-011-0399-x.

[3] STANAG 4487, Explosives, Friction Sensitivity Tests, NATO, Brussels, 2002.

[4] "NanoTechCenter" Ltd, CNTs "Taunit" and Synthesis Thereof, (2019). http://eng.nanotc.ru/producrions/87-cnm-taunit.

[5] N. Kubota, Propellants and Explosives, Wiley-VCH, Weinheim, 2006. http://doi.wiley.com/10.1002/9783527610105 (accessed May 11, 2016).

[6] N.V. Muravyev, THINKS – thermokinetic software, Moscow, 2016.

[7] T.B. Brill, P.E. Gongwer, Thermal Decomposition of Energetic Materials 69. Analysis of the kinetics of nitrocellulose at 50 °C-500 °C, Propellants, Explosives, Pyrotechnics. 22 (1997) 38–44. doi:10.1002/prep.19970220109.
